# Neurostructural correlates of strength decrease following total knee arthroplasty: A systematic review of the literature with meta-analysis

**DOI:** 10.17305/bjbms.2019.3814

**Published:** 2020-02

**Authors:** Armin H. Paravlic, Simon Kovač, Rado Pisot, Uros Marusic

**Affiliations:** 1 Science and Research Centre Koper, Institute for Kinesiology Research, Koper, Slovenia; 2 Valdoltra Orthopaedic Hospital, Ankaran, Slovenia; 3 Department of Health Sciences, Alma Mater Europaea – ECM, Maribor, Slovenia

**Keywords:** knee replacement, functional performance, rehabilitation, activation failure, total knee arthroplasty, quadriceps muscle weakness, TKA, VMA, CSA, MVS

## Abstract

Recent literature suggests that alterations in both neural and structural components of the neuromuscular system are major determinants of knee extensor muscle weakness after total knee arthroplasty (TKA). Therefore, the goal of this study was to investigate the maximal voluntary strength (MVS), voluntary muscle activation (VMA), and the cross-sectional area (CSA) of the muscle, up to 33 months after the TKA. We searched relevant scientific databases and literature for outcomes of interest, including quadriceps MVS, VMA, and CSA. Ten studies met the inclusion criteria and involved a total of 289 patients. The quality of the studies was evaluated by Methodological Index for Non-Randomized Studies (MINORS). Results showed that quadriceps MVS markedly declines in the early postoperative period, after which it slowly and linearly recovers over time. However, the same phenomenon was not observed for VMA and CSA, which were not significantly altered after the TKA. Furthermore, a meta-regression analysis revealed that the change in VMA accounted for 39% of the relative change in quadriceps strength (R^2^=0.39; p=0.015) in the early postoperative period. Patients treated with TKA had considerable weakness of the quadriceps muscle, which was detectable up to 3 months after surgery. Although the change in VMA largely explains quadriceps weakness, this change and CSA differences were not significant, suggesting that other neural correlates, such as hamstrings coactivation, might alter quadriceps muscle function. Thus, more attention should be paid to address VMA failure and coactivation of antagonist muscles. More comprehensive rehabilitation approaches may be required to target the whole neural circuit controlling the motor action.

## INTRODUCTION

Quadriceps muscle weakness represents a major determinant of physical function in patients who underwent total knee arthroplasty (TKA) [1,2]. Although TKA is a major surgical procedure, patients report significant pain relief and improved physical function after the surgery [3,4] and postoperative pharmacological and non-pharmacological treatments [5-10]. However, regardless of pain reduction in the early postoperative period, a substantial reduction in quadriceps strength persists a few months following TKA, which may be attributed to other reasons [6,11,12]. Accordingly, the cause of muscle weakness following TKA may be pain [13,14], joint injury (caused by chronic osteoarthritis [OA] and directly, by surgical trauma) [15–17], use of tourniquet during surgical procedure [18], postoperative knee swelling [19] and arthrogenic muscle inhibition [20,21].

It is well known that both central and peripheral neural factors influence the strength performance of an individual [22–24]. In a recent study by Morita and colleagues, muscle force decreased by 50% and 37.5% one, and two weeks after the unicompartmental knee arthroplasty, respectively [25]. In addition, the active brain region of the sensorimotor leg area narrowed [25]. However, the pain severity in the assessed knee two weeks postoperatively remained unchanged, suggesting that early postoperative muscle weakness was mostly influenced by the supraspinal pathways [25,26]. Also, there is evidence that failure to voluntarily activate muscles explains more than 60% of strength reduction following TKA, while atrophy contributes nearly 30% to this phenomenon [27]. When considered together, the muscle activation failure and muscle atrophy explain around 85% of strength reduction in the early postoperative period, without significant difference between pre- and postoperative pain level. Consequently, considerable attention was focused on investigating the neural and structural correlates of muscle weakness in the previous decade [28–31]. However, no study would comprehensively summarize the results of alterations in maximal voluntary strength (MVS), voluntary muscle activation (VMA) and cross-sectional area (CSA) of knee extensor muscles that occur after TKA surgery.

With the present systematic review, we aimed to explore the MVS of quadriceps muscle, VMA failure, and CSA of muscle loss, in patients who underwent TKA, up to 33 months postoperatively. In addition we analyzed correlation between MVS, VMA, and CSA in the early postoperative period.

## MATERIALS AND METHODS

### Search strategy

This systematic review and meta-analysis were undertaken following the Preferred Reporting Items for Systematic Reviews and Meta-Analyses (PRISMA) statement guidelines [32]. Thus, we conducted a systematic search of the literature for experimental trials studying neural and structural correlates of quadriceps strength after TKA in the adult population, published in peer-reviewed journals. To carry out this review, we searched English language literature in the MEDLINE/PubMed, Google Scholar, ScienceDirect, PEDro and SAGE Journals databases, published until 11^th^ September 2017. Additionally, we searched relevant journals such as the Journal of Arthroplasty; Knee; Knee Surgery, Sports Traumatology, Arthroscopy and The Bone & Joint Journal. Electronic databases were searched using the following keywords and their combinations: “total knee arthroplasty”, “knee replacement surgery”, “functional performance”, “functional impairment”, “quadriceps”, “voluntary muscle activation”, “central activation ratio”, “cross-sectional area”, “CSA”, “knee extensors muscles”, “muscle strength”, “torque”, “force”, “MVC”, “rehabilitation”. The reference lists of each included article were also viewed to identify additional relevant studies.

### Study selection

Eligible studies were selected by the following criteria:

(i) Population: Male and female adults of any age who underwent primary unilateral TKA for the treatment of knee osteoarthritis; (ii) Comparison: MVS, VMA, and CSA of the quadriceps muscle were compared pre- and postoperatively in various time points; and (iii) Outcomes: a) the isometric MVS which includes both the maximal voluntary isometric strength (MViC) expressed in absolute values, and relative MViC strength representing MViC normalized by kilograms of body weight, or body mass index (BMI); b) VMA; and c) CSA of the involved quadriceps muscle.

Exclusion criteria were the following: (a) studies that included patients who were scheduled for revision or bilateral TKA; and (b) studies from which we could not extract enough information to calculate effect sizes or include them in the analysis.

### Screening strategy

**FIGURE 1:**
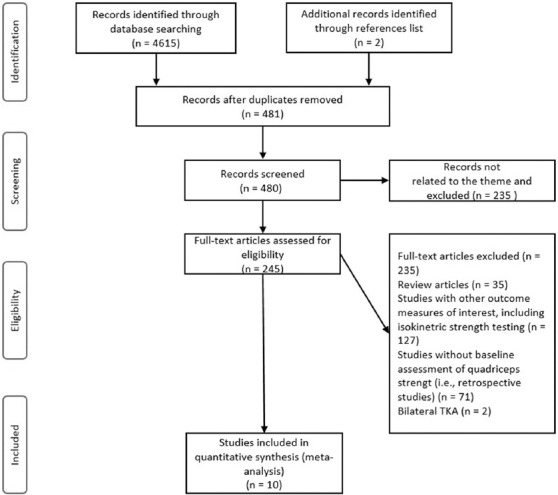
Flow diagram of the study selection process.

The study selection process illustrated in Figure 1. In brief, one researcher (AHP) performed the literature search, along with study identification, screening, quality assessment, and data extraction, after which another author (UM) checked all data independently. Following, detailed screening, the full texts of the remaining papers, that met the inclusion criteria were retrieved and included in the ongoing procedure and once again reviewed by the authors to reach a final decision on inclusion in the meta-analysis. In case of any disagreement between the reviewers, the third author (RP) was consulted. Finally, the reference lists from the retrieved manuscripts were also viewed for any other potentially eligible papers. If the full text of any paper was not available, the corresponding author was contacted by e-mail or via the ResearchGate platform.

### Data extraction

The Cochrane Consumers and Communication Review Group’s data extraction protocol was used to extract the participant information, including sex, age, sample size, training status, description of the intervention, study design, and study outcomes [33]. The first author undertook this extraction. The reviewer was not blinded to authors, institutions or journals. Corresponding authors were contacted or Web Plot Digitizer software (Version 3.10, Austin, TX, USA) was used to extract the necessary data from articles presenting the results in figures or graphs.

### Quality assessment

The first author (AHP) conducted the quality assessment. For observational or non-randomized studies, we used the 12-item Methodological Index for Non-Randomized Studies (MINORS) [34]. MINORS is a valid instrument designed to assess the methodological quality of non-randomized surgical studies, whether comparative or non-comparative. Each item was scored as “0” (not reported), “1” (not adequately reported), or “2” (adequately reported). The maximum score was 24 for comparative studies.

### Statistical analysis

The meta-analyses were performed using the Comprehensive Meta-analysis software (Version 2.0, Biostat Inc., Englewood, NJ, USA). The effect size is calculated according to the following formula: 
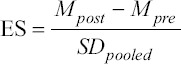
 where ES represents the effects size, *M_post_* is mean value after surgery (POST), *M_pre_* is mean value before surgery (PRE) and *SD_pooled_* represents pooled standard deviation (SD). According to Hedges, this formula was adjusted for sample size: 
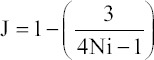
 where J is the adjusted ES and Ni is the total sample size of the intervention group minus one [35]. We calculated the mean differences and 95% confidence intervals (CIs) for the included studies. The I^2^ measure of inconsistency was used to assess between-study variability; values of 25%, 50% and 75% represent low, moderate and high statistical heterogeneity, respectively [36]. Although the heterogeneity of the effects in this meta-analysis ranged from 0% to 93.7%, we decided to apply a random-effects model in all comparisons, to determine the pooled effect of TKA on MVS, VMA and CSA [37,38].

If one study reported both absolute values, i.e., non-normalized data, and normalized data for muscle strength, only the non-normalized data were taken into consideration. Also, we performed univariate meta-regression to estimate the influence of VMA on muscle strength reduction in the early days following TKA. In studies where there were multiple assessments of a single population that occurred in the same category for time period (e.g., when summarizing effects of one and one and a half as one-time period for meta-regression only), only one assessment point was included to avoid an individual subject being represented twice in the same meta-analysis. Further, we conducted a sub-analysis to investigate the magnitude of the observed effect of the rehabilitation treatment, i.e., outpatient professionally-guided rehabilitation (OPGR) vs. usual care (UC). The significance level of p ≤ 0.05 was used for all analyses.

The publication bias was assessed by examining the asymmetry of the funnel plots using Egger’s test, and significant publication bias was considered if the p < 0.10. The magnitude of effect was interpreted using the following criteria: trivial (< 0.20), small (0.21–0.60), moderate (0.61–1.20), large (1.21–2.00), very large (2.01–4.00) and extremely large (> 4.00).[39]

## RESULTS

**FIGURE 2:**
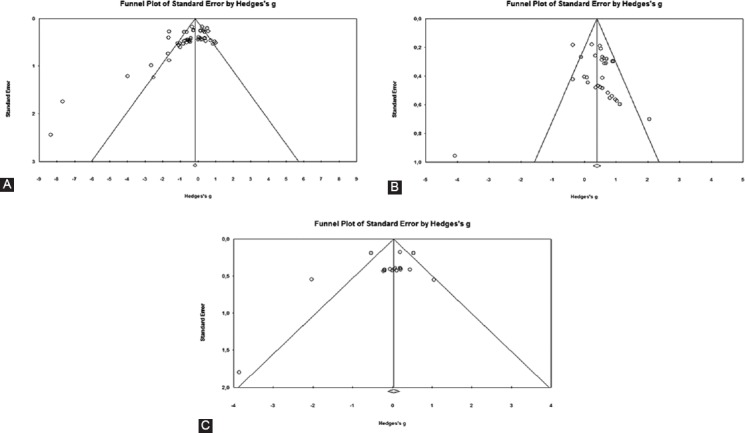
Funnel plots of the standard differences in means vs. standard errors for (A) maximal voluntary strength, (B) voluntary muscle activation and (C) cross-sectional area of the quadriceps muscle.

The Egger’s test indicated publication bias for all analyses (all values of p ≤ 0.075) (Figure 2).

### Study selection

A total of 4,615 articles were identified by the literature search (Figure 1.). However, 235 studies remained, following the elimination of duplicates and exclusion of articles based on the title and abstract screening. These studies were then evaluated and, after the final screening process, 10 studies were included in the systematic review and meta-analysis.

#### Study characteristics

After the study selection process, 10 eligible articles were found (Table 1). Table 1 presents details about study sample, measures, results, and additional comments. All included studies investigated MVC, among which eight, and four studies additionally investigated VMA [27–30,40–43] and CSA [5,27,30,44], respectively. When the studies presented different subgroups of patients, e.g., receiving different pre- and postoperative physical therapy regimens, these were considered as different pre-post comparisons. Notably, the included studies reported measures of quadriceps muscle strength differently, whereby seven and three of the included studies investigated MViC, [5,28,41–45] and MViC normalized to kg of body weight [29,40] or BMI [27], respectively. Seven studies had more than one measurement assessment following the surgery, in periods ranging from three and a half weeks, up to 33 months[5,29,40,42–45]. In all included studies, the first unilateral TKA was performed primarily because of OA. Three studies did not report the surgical approach used [5,40,45]. Other studies used standard medial parapatellar approach [27,29,44], and medial arthrotomy [42], while in one study both anterior linear and medial parapatellar approaches were used [43]. Regarding postoperative rehabilitation, the most commonly used physical therapy treatment was assessed. However, in two studies a preoperative exercise program was implemented [42,44]. Four studies carried out the postoperative professionally-guided, progressive rehabilitation treatment[5,29,30,40], among which one study used neuromuscular electrical stimulation (NmES) in addition to OPGR, to investigate its influence on early postoperative functional outcomes following TKA [29].

**TABLE 1:**
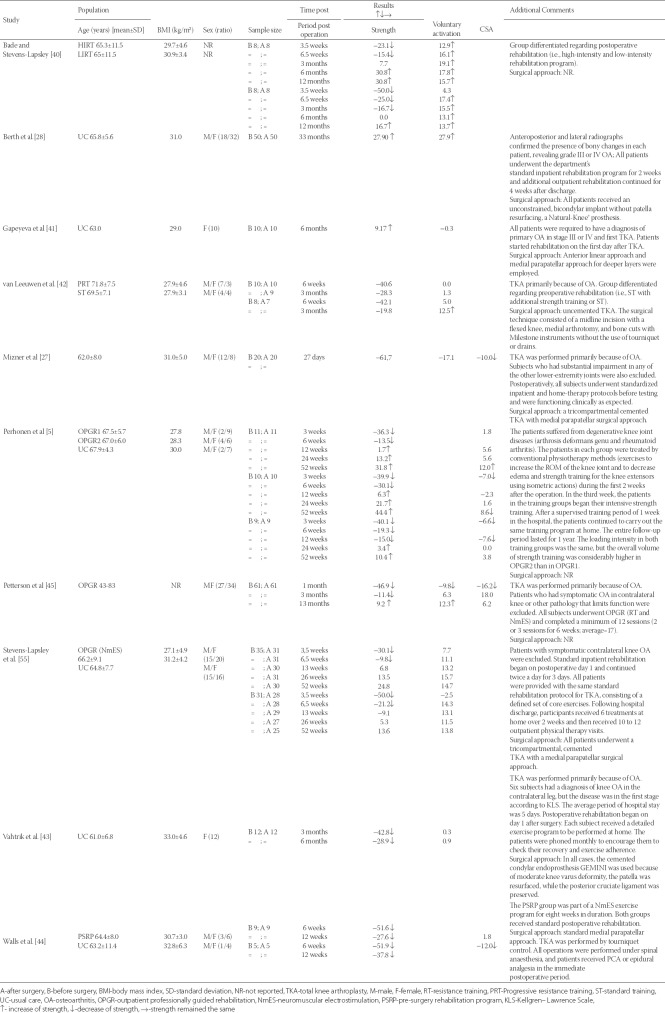
Systematic review and characteristics of included studies selected for meta-analysis and relevant outcomes

#### Subject characteristics

The pooled sample size of the 10 studies included 289 subjects, while the sample size of the individual studies ranged from 5 to 61 subjects per group (mean: 18 subjects). Information about sex ratio was provided in all studies, with a total of 61% female subjects. Age was provided in all studies, with a preoperative mean of 65.6 years (range 61.0 to 71.8 years). BMI was reported in almost all studies, except one with a mean value of 29.9 kg/m^2^ (range from 27.1 to 33.0 kg/m^2^) [45].

### Quality assessment

The mean MINORS score for the included observational studies was 18.6 (range 14 to 22 points; Table 2). All reviewed studies received a maximum of 2 points for the following items: a clearly stated aim, the inclusion of consecutive patients, the prospective collection of data, and adequate statistical analyses of data. In addition, only two studies reported unbiased assessment of the study endpoint [29,44]; one study had more than 5% subject loss during the follow-up period [40]; five studies did not have prospective calculation of the study size [5,27,28,30,44]; one study did not have a comparison group at all [27]; and one study reported inconsistency of the groups in the baseline [28].

**TABLE 2:**
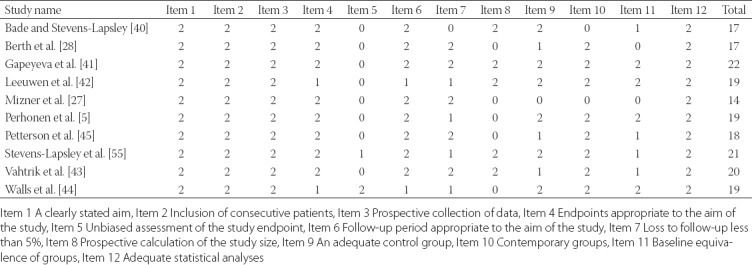
Quality assessment of included studies by using Methodological Index for Non-Randomized Studies (MINORS)

### Overall findings

#### Maximal Voluntary isometric Contraction Strength following total knee arthroplasty surgery

**FIGURE 3:**
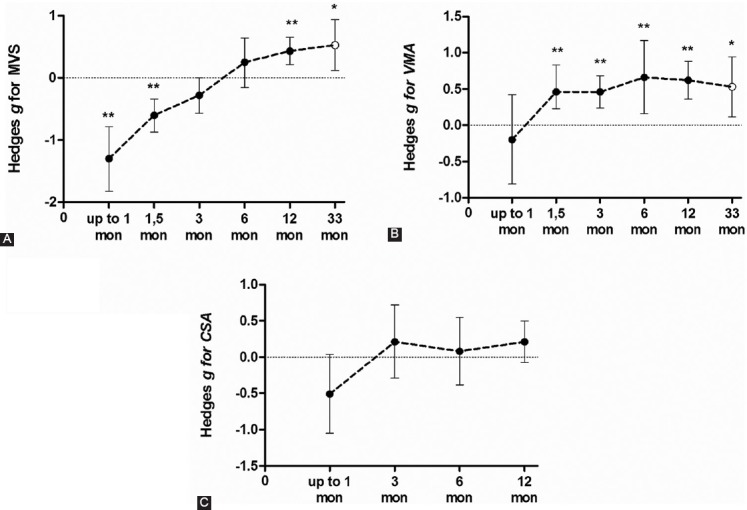
Summarized effect of more than one study (closed circle) and one study only (open circles) demonstrating time course of (A) quadriceps muscle maximal voluntary strength (MVS) recovery, (B) voluntary muscle activation level (MVA); and (C) Cross-Sectional Area (CSA) or different time points comparing pre- to post-surgery values. Data were presented as effect size and its lower and upper limits of 95% confidence interval.

Five studies (nine ESs) assessed the MVS one-month after surgery, and showed the most likely major harmful effect on MVS (ES = -1.30; 95 % CI -1.82 to -0.79; p < 0.001; *I^2^* = 63.6%) (Figure 3A). Following this considerable decline, MVS starts to recover linearly. After one and a half months (small ES = -0.60; 95 % CI -0.87 to -0.34; p < 0.001; df = 10; *I^2^* = 5.0%) and three months postoperatively (small ES = -0.28; 95 % CI -0.57 to -0.00; p = 0.054; df = 12; *I^2^* = 41.9%), strength was still lower when compared to preoperative values. Moreover, there was no significant difference six months after surgery (small ES = 0.25; 95 % CI -0.15 to -0.64; p = 0.217; df = 9; *I^2^* = 51.6%) as compared to preoperative values, suggesting that patients regained their strength to the baseline (preoperative) values. One-year (small ES = 0.43; 95 % CI 0.21 to -0.65; p < 0.001; df = 7; *I^2^* = 0%) and 33 months after surgery (small ES = 0.53; 95 % CI 0.12 to - 0.94; p = 0.012; df = 0; *I^2^* = 0%), MVS was significantly higher when compared to preoperative values.

#### Quadriceps Voluntary Muscle Activation following total knee arthroplasty surgery

**FIGURE 4:**
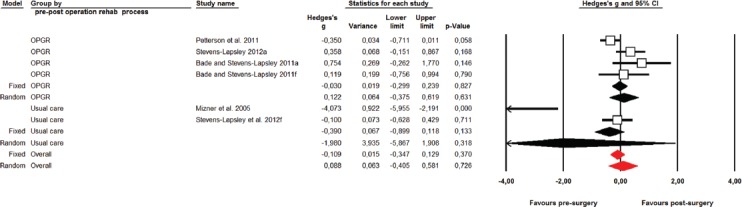
Effects of outpatient professionally guided practice vs. usual care rehabilitation practice on voluntary muscle activation following TKA.

Four studies (six ESs) assessed the VMA one-month after surgery. The summarized effect showed the possibly harmful and trivial effect on VMA (ES = -0.20; 95 % CI -0.81 to -0.42; p = 0.533) (Figure 3B). Due to significantly large heterogeneity of the observed effect (Q = 25.03; p < 0.001; *I^2^* = 80.0%), an additional sub-analysis was conducted. Namely, the effects were determined based on the postoperative rehabilitation process (OPGR vs. UC) on the magnitude of knee extensors’ VMA (Figure 4). Thus, UC showed a likely large harmful effect (ES = -1.98; 95 % CI -5.87 to 1.91; p = 0.318; df = 1; *I^2^* = 93.7%), while OPGR showed possibly trivial effect (ES = 0.12; 95 % CI -0.38 to 0.62; p = 0.631; df = 3; *I^2^* = 60.8%) suggesting that OPGR programs might have some positive effects on VMA preservation after TKA surgery. However, due to high methodological heterogeneity among the included studies and in individual subjects (pre-and postoperative measurements), no firm conclusion could be drawn.

One and a half months after surgery, very likely small beneficial effect was observed on the VMA of the involved quadriceps muscle (ES = 0.53; 95 % CI 0.23 to -0.83; p < 0.001; df = 5; *I^2^* = 0%). In the following months, the VMA significantly recovered in a linear fashion. Thus, very likely small beneficial effect was observed after three months (ES = 0.46; 95 % CI 0.24 to 0.68; p < 0.001; df = 8; *I^2^* = 0%), while after six months (ES = 0.66; 95 % CI 0.16 to 1.17; p = 0.010; df = 5; *I^2^* = 55.4%), and twelve months (ES = 0.62; 95 % CI 0.36 to 0.88; p < 0.001; df = 4; *I^2^* = 0%), the effect was moderately beneficial. Thirty-three months after surgery, a likely small beneficial effect was observed (ES = 0.53; 95 % CI 0.12 to 0.94; p = 0.012; df = 0; *I^2^* = 0%).

#### The Cross-Sectional Area of quadriceps muscle of the affected leg

Three studies (five ESs) assessed the MVS one-month after surgery and showed a likely harmful effect on the CSA of the quadriceps muscle (ES = -0.51; 95 % CI -1.05 to 0.04; p < 0.067; *I^2^* = 63.8%) (Figure 3C). Following this early postoperative decline, the CSA starts to recover. Three months (small ES = 0.21; 95 % CI -0.29 to 0.72; p = 0.410; df = 5; *I^2^* = 53.9%), six months (trivial ES = 0.08; 95 % CI -0.38 to 0.55; p = 0.723; df = 2; *I^2^* = 0%), and one-year (small ES = 0.21; 95 % CI -0.07 to 0.50; p = 0.140; df = 3; *I^2^* = 0%) postoperatively, the CSA was not significantly different when compared to preoperative values.

#### Meta-Regression analysis

The conducted univariate meta-regression analysis of eight included ESs revealed that the change of VMA accounted for 39% of the relative change in quadriceps strength of the affected leg after one to one and a half months after surgery (Z = 2.44; R^2^ = 0.39; p = 0.015).

## DISCUSSION

In this systematic review and meta-analysis, we have quantified the data from the available literature to determine the time course of strength, VMA and CSA reduction, and recovery in patients with chronic OA who underwent TKA surgery. Therefore, we can make the following statements:

1) In the early postoperative days, the quadriceps strength markedly declines, after which it slowly recovers linearly over time, thus being significantly greater one year after surgery.
2) The VMA was lower one-month after surgery; however, the observed decline was not significant due to the significant heterogeneity between the included studies regarding the use of outpatient professionally-guided rehabilitation or only the usual care.
3) Patients who underwent OPGR showed a considerably lower magnitude of VMA decline when compared to those who underwent the UC rehabilitation program, suggesting that a more progressive, holistic and professionally supervised rehabilitation may have more beneficial effects on VMA conservation following TKA.
3) The CSA of the operated leg/quadriceps muscle was negatively affected one-month after surgery; however, it was not significantly altered in any following period after surgery, when compared to preoperative values. Insignificant alterations occurred most probably due to high heterogeneity among the analyzed studies (see CIs on Fig 3C).
4) A meta-regression analysis showed that the change of VMA accounted 39% of the relative change in quadriceps strength of the affected leg up to one and a half months after surgery, suggesting that more attention should be addressed to VMA in early rehabilitation of TKA patients.

Previously, it was shown that quadriceps strength is significantly affected by TKA surgery, and the quadriceps weakness persists up to 3 years after TKA when compared to healthy age-matched individuals [46]. However, in this study, we aimed to compare pre- and postoperative strength values in patients in whom the period needed to regain preoperative knee extensor muscle strength was approximately six months, while significant improvement should be expected one-year after surgery. A recent meta-analysis investigated the benefits of OPGR and UC rehabilitation programs and showed a small to moderate, short-term beneficial effect that favors OPGR, with no long-term benefit in one-year postoperative period [47]. Different protocols of muscle strengthening have been used to counteract quadriceps weakness, such as progressive muscle strengthening [30], both high-intensity [40] and high-volume training [5], whole-body vibration exercise [48], aquatic training [49], neuromuscular electrical stimulation and the most recent non-physical approaches like action observation and motor imagery [50–52]. However, it is possible that current rehabilitation protocols may be inadequate, considering the type of intervention [52], its duration, included exercises, intensity volume, etc. [53]. Voluntary muscle activation explained almost half of quadriceps weakness one and a half months after surgery; however, a pooled effect did not reveal a significant MVA decrease. Interestingly, MVA showed significant recovery and reached the plateau already one and a half months after surgery, while the quadriceps muscle CSA was not significantly altered in any of the postoperative periods. This suggests that other factors rather than MVA and quadriceps CSA limit maximal strength outputs of knee extensor muscles.

Accordingly, a variety of factors could alter the functional performance of knee extensors such as pain [13,14], joint damage [15–17], use of tourniquet during operative procedure [18], inflammation [54] and postoperative knee swelling [19], eventually inducing arthrogenic muscle inhibition (AMI) [20,21]. While most of the agents mentioned above disappear within the acute postoperative period (up to 3 months), persistent quadriceps weakness may be explained by antagonist muscle activity during both open and closed chain movement execution [55]. Intra- and intermuscular coordination are essential factors that may improve movement efficiency by increasing joint stabilization and prevent injury; however excessive coactivation may impair movement execution and cause agonist weakness [56]. For example, patients with severe OA have a higher coactivation pattern of muscles surrounding the knee joint during walking and consequently lower functional performance as compared to healthy age-matched subjects. The coactivation index is calculated as a ratio between peak hamstrings electromyography (EMG) during quadriceps MViC and peak hamstrings EMG during hamstrings MViC. This index turned out to be higher in operated leg compared to the non-operated leg (144.5% elevation) one month after surgery. Similar patterns of antagonist coactivation during knee extensors MViC persisted in both follow-up periods (three and six months after surgery), however without statistically significant difference[55]. Knowing that antagonistic muscle activity increases when the complexity of the movement rises (e.g., during closed chain, weight-bearing exercise), quadriceps function is more affected leading to greater difficulty in performing everyday activities such as walking or standing up from the chair, thus affecting patients’ overall functionality and quality of life [57]. A recent comprehensive review of available literature found motor imagery (MI) practice to have moderate beneficial effects on strength gains, regardless of the cortical representation of trained muscle, suggesting that both large and small cortically represented muscles can almost equally benefit from MI practice [58]. The underlying mechanisms of the observed strength gains might be explained by alteration of both central and peripheral levels of muscle action control [59,60], with evidence of higher agonist activation [61] followed by antagonistic muscle inhibition during agonistic muscle action [62]. Therefore, incorporating MI practice in early stages of injury or surgical rehabilitation should be considered, when overt movement is restricted [50,58,61].

### Limitations

This study had several limitations. Firstly, a methodological heterogeneity among the included studies regarding experimental design, use of different post-rehabilitation protocols, and measurement assessment of voluntary muscle activation (e.g., superimposed burst technique or interpolated twitch technique) make it difficult to compare the effect between studies. However, we lowered the possible bias of quantifying the effect using within-subject comparison and calculating the effects as standardized mean difference, adjusted for sample size. There were limitations in the external validity as well: almost all the subjects included were affected by severe OA or were scheduled for primary TKA, and inclusion into the original study was mainly limited to those subjects with a BMI of less than 35 kg/m^2^. Therefore, no comparison could be made between different types of surgery types (TKA vs. unicompartmental knee arthroplasty), as well as between obese, overweight and healthy weight subjects. Finally, the publication bias results indicated the presence of bias. It is possible that some studies may not have been published, due to null or negative results, reducing the generally positive effect of TKA practice on strength, voluntary activation level and CSA of knee extensor muscles.

## CONCLUSION

A considerable decrease in strength of the involved quadriceps muscle following TKA, lasting several months after surgery, was observed. Interestingly, voluntary muscle activation was significantly higher compared to preoperative values, already one and a half months after surgery, while insignificant alterations were observed in CSA after surgery. The present findings suggest that other neural correlates, such as antagonistic hamstring activation, alter quadriceps muscle function during both open and closed chain activities. Future studies should specifically target bilateral strengthening of the quadriceps muscles, focusing on both the functional (quadriceps/hamstrings) and lateral (operated/non-operated leg) strength symmetry, respectively. Interventions that incorporate both the central (neural circuits controlling motor action) and peripheral (executing motor action) components of movement execution would be desirable.
